# Trajectories of Childhood Housing Insecurity and Links to Emerging Adulthood Depression: A Repeated Measures Latent Class Approach

**DOI:** 10.1002/jcop.70078

**Published:** 2026-01-06

**Authors:** Katherine Marçal

**Affiliations:** ^1^ School of Social Work Rutgers University New Brunswick New Jersey USA

**Keywords:** depression, emerging adulthood, housing insecurity, repeated measures latent class analysis

## Abstract

Nearly one in three emerging adults experience depression. Past exposure to socioeconomic hardship increases risk, but little research investigates how disparate trajectories of childhood housing insecurity influence mental health in the transition to adulthood. Repeated measures latent class analysis in a sample of youth from families born in 20 large American cities (*N* = 2,239) identified four unique trajectories of housing insecurity from infancy to adolescence characterized by (1) consistently low housing insecurity (75.4%), (2) early childhood housing insecurity that subsequently stabilizes (5.4%), (3) moderate housing insecurity increasing slightly in adolescence (16.6%), and (4) severe early childhood insecurity that ultimately declines but remains elevated compared to other groups (2.6%). The fourth subgroup displayed significantly elevated risk for depression in emerging adulthood compared to other groups. Findings suggest that severe housing insecurity in early childhood, even with relative improvement over time, threaten long‐term mental health. Addressing early housing insecurity offers promise for preventing depression and promoting healthy development in the transition to adulthood.

Emerging adulthood is a high‐risk period for depression (Arnett et al. 2014). The transitional nature of this developmental period in which many begin to live independently and form new relationships can leave youth vulnerable to mood disorders which may persist across adulthood (Carey et al. 2024). The prevalence of depression has increased in recent years, with the largest jump occurring among young adults age 18–35 (Greenberg et al. 2021). Childhood housing insecurity—in which children lack continuous access to safe, stable, affordable living arrangements—is an established risk factor for subsequent depression, but little research examines this relationship from childhood into emerging adulthood. Housing cost burden, eviction, moving in with family or friends, and shelter stays can have long‐term impacts on mental health that impede healthy development. The diverse and dynamic nature of housing insecurity experiences among families with children require a person‐centered approach to more closely probe the link with depression in the transition to adulthood.

## 1 Depression in Emerging Adulthood

Emerging adulthood, a transitional developmental phase from the late teens through the twenties, is characterized by rapidly evolving life circumstances, expanding independence and responsibilities, and relationship exploration and formation (Arnett 2000). Along with continued brain maturation, this life stage brings high risk for relational and financial precarity as youth explore relationship, education, and career options (Arnett et al. 2014; Murray and Arnett 2019). The completion of specific developmental tasks in emerging adulthood is associated with improved long‐term outcomes (Roisman et al. 2004), and mental health may be dependent on a combination of heritable risk from parents (Ksinan and Vazsonyi 2019), youth characteristics such as race and gender (Albert 2015; Wade et al. 2002), and exposure to adversities (Evans and Kim 2012). The transitional, exploratory nature of emerging adulthood combined with rapid social emotional development (Hochberg and Konner 2020) can lead to divergent mental health status as a function of personal histories and current supports (Wood et al. 2018).

Depression is major health outcome of concern in emerging adulthood (Arnett et al. 2014). A recent nationally representative survey found a depression rate of 29% among young adults aged 18–25 years—substantially higher than the depression rate among teens age 14–17 (15%; Weissbourd et al. 2023) and the lifetime depression rate among U.S. adults (Lee et al. 2023). A growing body of research links depression in emerging adulthood with risk for long‐term mental disorder. Higher levels of depression in the transition to adulthood have been associated with subsequent lower career and overall life satisfaction (Howard et al. 2010) as well as lower social status attainment defined by levels of educational attainment, employment, living independently, and forming stable intimate relationships (Wickrama et al. 2008). Failure to reduce risk for depression in emerging adulthood may have enduring consequences for overall well‐being across the life course.

## 2 Childhood Housing Insecurity and Emerging Adulthood Depression

Emerging research suggests childhood housing insecurity may relate with risk for depression in emerging adulthood. Children exposed to housing insecurity may experience disrupted routines and relationships (Larsen and Jordan 2020; Mayberry et al. 2014), high levels of individual and caregiver stress (Bills et al. 2019; Marçal 2022b), aggressive parenting or maltreatment (Chandler et al. 2022; Marçal 2018, 2022a), and exposure to precarious, chaotic, or dangerous conditions (Coley et al. 2013, 2015; Evans and Kim 2012) that impede healthy emotional development. A large body of research links childhood housing insecurity with concurrent or short‐term emotional and behavioral problems (e.g. Buckner 2008; Larsen and Jordan 2020); more recent research further suggests an association between childhood housing insecurity and long‐term mental health outcomes in adolescence and the transition to adulthood. In a sample of children from large American cities drawn from the Future of Families and Child Well‐Being Study (FFCW), housing insecurity (including inability to make rent payments, doubling up, eviction, or homelessness) at age five has been consistently associated with adolescent depressive symptoms 10 years later (Hatem et al. 2020; Marçal et al. 2023; Marçal and Maguire‐Jack 2021). A subsample analysis using survey and neuroimaging data from the same study (Hardi et al. 2023) found that household instability in early childhood was associated with more symptoms of depression in young adulthood via white brain matter network organization. A composite measure of childhood socioeconomic adversity, which included housing insecurity, was associated with higher levels of depression across adolescence and into emerging adulthood in a sample of rural youth followed over 10 years (Wickrama et al. 2008). A prospective cohort study of children from the southeastern United States investigated the relationship between childhood housing insecurity—including frequent moves, reduced standard of living, forced displacement, and foster care stays—and short‐ versus long‐term mental health (Keen et al. 2023); analyses identified a significant link between childhood housing insecurity and concurrent depression and anxiety, and a significant link between childhood housing insecurity and subsequent adult depression between ages 19 and 30.

The nature of housing insecurity among families with children is dynamic, complicating efforts to understand its true impact on outcomes and target prevention strategies. Severe housing crises like eviction and homelessness are relatively rare and short‐lived (Eviction Lab 2022; Henry et al. 2023), whereas periods of inadequate or precarious housing such as cost burden, frequent moves, or doubling up may occur intermittently or persist as a function of family resources and economic factors (Joint Center for Housing Studies of Harvard University 2011, 2023; Kids Count Data Center 2022). Housing insecurity may recur for vulnerable families or stabilize over time. Fewer studies examine associations of divergent childhood trajectories of housing insecurity with subsequent mental health outcomes. A recent exception (Pierce et al. 2024) investigated housing from age one to 15 among a disproportionately low‐income U.S. sample of families with children (*N* = 4,714); the study identified three distinct trajectories of housing insecurity from age one to 15 along a continuum of severity (low, moderate, and high). The most securely housed group experienced lower levels of depression compared to the other groups at age 15, but the study did not investigate more distal impacts on depression in the transition to adulthood. More research is needed to understand how divergent trajectories of housing insecurity in childhood may be associated with divergent emerging adult outcomes.

## 3 Developmental Timing of Childhood Housing Insecurity

In addition to differing frequencies of housing insecurity, children may experience instances of housing insecurity in different developmental stages. A growing body of research investigates the significance of the developmental timing of housing insecurity for long‐term outcomes. Many studies point to early childhood as a significant period during which children face high risk for housing insecurity as well as high vulnerability to developmental threats from housing insecurity (Coley et al. 2015; Hatem et al. 2020; Leifheit et al. 2020). The time and cost demands of young children increase families' risk for housing cost burden, precarious or dangerous housing arrangements, and displacement through eviction or forced moves. More than five percent of U.S. children under age five experience eviction each year, and one in three people in households who receive an eviction filing are under age 15 (Graetz et al. 2023). Families experiencing homelessness have younger children on average than families in poverty or U.S. families overall, and approximately half of children in homeless shelters are under age five (Henry et al. 2023).

At the same time, lack of stable housing during early childhood has significant negative long‐term impacts. Stability and predictability in the immediate environment are crucial in early childhood (Shonkoff 2010), and housing insecurity during this period threatens healthy socioemotional development (Foster et al. 2024). Unpredictability in the home environment, which characterizes many experiences of housing insecurity, may impact very young children's abilities to process environmental cues and shape future cognitive and emotional responses to change (Ugarte and Hastings 2023). Consequently, a number of studies confirm that early childhood housing insecurity is associated with poor mental health outcomes at least into late childhood and adolescence, although evidence of continuing impact into emerging adulthood is lacking. Exposure to residential instability in the first 2 years of life has been associated with increased subsequent child emotional and behavior problems at age 9 in a sample of Australian children (Rumbold et al. 2012), and exposure to broader housing insecurity at age three has been associated with emotional and behavior problems at age 15 in the American FFCW sample (Marçal 2022c; Marçal et al. 2023). Little research, however, examines the impact of early housing insecurity on mental health beyond adolescence and into the transition to adulthood.

Additional research suggests that, like early childhood housing insecurity, chronic housing insecurity across childhood may pose unique risks to long‐term mental health. Chronic or repeated exposures to environmental adversity threaten healthy development across multiple domains, including emotional and behavioral well‐being (Shonkoff 2010). The allostatic load, or cumulative exposure to chronic stress, significantly increases risk for depression across the life course (Guidi et al. 2020). Among children, repeated or high levels of exposure to inadequate housing has been found to increase a range of physiological markers and processes associated with chronic stress (Evans and Kim 2012). Cumulative childhood exposure to the conditions of poverty, characterized in part by inadequate housing, has also been linked to higher emotional and behavior problems at age 24 (Evans and De France 2022). Taken together, this growing body of research suggests that both early childhood housing insecurity as well as persistent housing insecurity across childhood may be linked with elevated risk for depression in the transition to adulthood, though studies investigating this link that capture a wide range of childhood housing experiences are lacking.

## 4 Present Study

The present study investigated whether childhood trajectories of housing insecurity were associated with depression in emerging adulthood. Specifically, the study addressed the following two research questions: 1) Are there distinct subtypes of childhood housing trajectories from infancy to adolescence? and 2) Are subtypes of childhood housing trajectories associated with differential risk for depression in emerging adulthood? Findings will inform understanding of experiences of childhood housing insecurity and identification of youth at greatest risk for long‐term mental disorder.

## 5 Methods

### 5.1 Data and Sample

Data for the present study came from the Future of Families (formerly “Fragile Families”) and Child Well‐being Study (henceforth, FFCWS). FFCWS used a stratified clustered random sampling design to select 20 large cities in the U.S. with varying economic contexts and levels of welfare generosity, hospitals within cities, and births within hospitals that occurred 1998–2000, with an intentional oversample of births to unmarried parents. Mothers were recruited and interviewed in hospitals shortly after giving birth, with fathers recruited and interviewed either in the hospital or as soon as possible thereafter. Primary caregivers—usually biological mothers, but at times biological fathers or others—were followed up at one, three, five, nine, and 15 years. The most recent wave of data collection, Year 22, was collected when study focal children were roughly 22 years old and included interviews with both the child (now emerging adult) as well as the Year 15 primary caregiver; approximately one‐third of families in the original baseline sample were lost to follow‐up, although the Year 22 sample did not significantly differ from the baseline sample on key sociodemographic characteristics (Bendheim‐Thoman Center for Research on Child Wellbeing & Columbia Population Research Center 2024; Princeton University 2024). The analytic sample for the present study was limited to households in which the focal child lived with the biological mother or father at least half the time through Year 15, and in which the emerging adult participated in the Year 22 interview (*N* = 2,239; Table 1).

**Table 1:** Sample description.

	*M* (SD)	*N* (%)
**Outcome variable**		
Emerging adulthood depression		619 (39.28%)
**Latent class indicators**		
Housing insecurity frequency^a^		
Year 1		
0		1,750 (78.16%)
1		374 (16.70%)
2+		115 (5.14%)
Year 3		
0		1,817 (81.15%)
1		328 (14.65%)
2+		94 (4.20%)
Year 5		
0		1,811 (80.88%)
1		343 (15.32%)
2+		85 (3.80%)
Year 9		
0		1,721 (76.86%)
1		440 (19.65%)
2+		78 (3.48%)
Year 15		
0		1,800 (80.39%)
1		363 (16.21%)
2+		76 (3.39%)
**Covariates**		
Mother's race		
White		534 (24.05%)
Black		1,122 50.44%)
Hispanic		506 (22.08%)
Other		77 (3.43%)
Father's race		
White		477 (21.13%)
Black		1,165 (52.73%)
Hispanic		510 (22.40%)
Other		87 (3.74%)
Mother's age at birth	25.49 (6.08)	
Parents married at birth		619 (27.65%)
Childhood poverty		1,405 (62.75%)
Caregiver depression		1,487 (66.41%)
Youth gender		
Male		1,092 (48.77%)
Female		1,147 (51.23%)
Lives with parent^b^		1,265 (56.50%)
Currently working^b^		1,434 (64.05%)
Currently in school^b^		648 (28.94%)
Childhood anxiety/depression	49.96 (6.75)	
Current housing insecurity frequency^a^,^b^		
0		1,738 (78.36%)
1		409 (18.15%)
2+		92 (3.50%)

^a^ Number of housing hardships experienced in the past year at each wave.

^b^ Measured at Year 22.

## 6 Measures

### 6.1 Latent Class Indicators

Latent classes were identified using repeated measures of housing insecurity at the Years 1, 3, 5, 9, and 15 follow‐up waves. At each wave, the child's primary caregiver (biological mother or father) reported whether or not they had experienced a missed rent or mortgage payment, eviction, doubling up with others, or a single night of literal homelessness (living on the street, in a car, or somewhere else not meant for human habitation) due to inability to afford housing in the previous 12 months. Responses (1 = yes, 0 = no) for all four items were summed at each wave such that higher scores indicated more instances of housing insecurity. Total scores ranged from 0 to 4, but scores at the higher end were collapsed due to the right skewed nature of responses. Final scores were categorized as 0 = no housing hardships, 1 = 1 housing hardship, and 2+ = 2 or more housing hardships at each of the five waves.

### 6.2 Distal Outcome

The outcome variable was emerging adulthood depression measured at the Year 22 interview using the Composite International Diagnostic Interview‐Short Form (CIDI‐SF; Kessler et al. 1998). Youth, who were roughly 22 years old, answered a series of questions about the severity and frequency with which they experienced symptoms of depression such as loss of interest or enjoyment, low mood, weight and sleep changes, and suicidal thoughts. Scores were calculated by summing endorsed symptoms, with a cutoff imposed to create a dichotomous indicator of likely major depressive disorder consistent with diagnostic criteria in the Diagnostic and Statistical Manual of Mental Disorders, Fourth Edition (DSM‐IV; (American Psychiatric Association 2000; Walters et al. 2002).

### 6.3 Covariates of Latent Class Membership

A number of household and family characteristics were included as covariates of latent class membership. *Mother's race* and *Father's race* were categorized as White, Black, Hispanic, and Other. *Mother's Age at Birth* was the mother's age in years at the time of the study focal child's birth. *Parents Married at Birth* was a dichotomous indicator of whether or not the biological parents were married at the time of the focal child's birth. *Childhood poverty* indicated whether or not the child's primary caregiver (biological mother or father) reported a household income below the federal poverty line at any of the Years 1–15 interviews, suggesting the child had spent time in poverty.

### 6.4 Covariates of Distal Outcome

A second set of covariates were included in analyses of the distal outcome, emerging adulthood depression. Self‐report *youth gender* was dichotomized as male and female. Youth reported whether they currently *lived with parent(s)* at Year 22, whether they were *currently working*, and whether they were *currently in school* (1 = Yes, 0 = No). *Current housing insecurity* indicated the extent of housing insecurity reported by emerging adults at the Year 22 interview; youth responded to the same housing items administered to caregivers in earlier waves about whether or not they had experienced a missed rent payment, eviction, doubling up, or homelessness in the past 12 months; responses were summed and categorized as 0 = no housing hardships, 1 = 1 housing hardship, and 2+ = 2 or more housing hardships. *Caregiver depression* was a dichotomous measure that indicated whether the primary caregiver at each wave—either the biological mother or father—had ever displayed major depression using the same tool and scoring scheme as emerging adulthood depression described above. *Childhood anxiety/depression* captured the extent to which youth had displayed problematic depressive or anxious behaviors in childhood using the anxious/depressive subscale from the Child Behavior Checklist (CBCL; Bendheim‐Thoman Center for Research on Child Wellbeing & Columbia Population Research Center 2018). Caregivers reported the extent to which children displayed behaviors or characteristics such as crying, clinginess, fearfulness, and sadness at the Years 3, 5, 9, and 15 interviews (the CBCL was not administered at Year 1). Responses were averaged and converted to standardized scores at each wave; to avoid multicollinearity issues, standardized scores from all four waves were then averaged to create a single average score ranging from 0 to 100, with higher scores indicating worse anxious/depressive behavior problems across childhood. The Cronbach's alpha for all items was 0.81, indicating high inter‐item reliability across waves.

### 6.5 Analytic Approach

Childhood housing trajectories were assessed using a repeated measures latent class analysis (RMLCA). Repeated measures of a single variable—in this case, housing insecurity—were used as indicators for a latent class analysis that captured unobserved subgroups of individuals' housing experiences over time. This person‐centered approach used the maximum likelihood estimator to fit and compare the 1‐ through 5‐class solutions. Model fit was assessed by comparing Akaike Information Criterion (AIC), Bayesian Information Criterion (BIC), and entropy values. Lower AIC and BIC values indicate better fit, whereas entropy values close to 1 indicate strong class separation (Celeux and Soromenho 1996; Hu and Bentler 1999). The optimal class solution was selected based on a range of criteria, including fit indices, parsimony, and interpretability (Nylund et al. 2007).

Analyses investigated the association between covariates and likely latent class membership by first assigning individuals to latent classes using their maximum posterior probabilities (Nagin and Group‐Based 2005), and then including covariates as auxiliary variables; multivariate logistic regression treated likely latent class membership as a categorical dependent variable with covariates as independent variables (Asparouhov and Muthén 2013).

Outcome analyses investigated whether probabilities of emerging adulthood depression differed by latent class membership. The unadjusted distribution of the probability of depression was estimated by likely class membership (Lanza et al. 2013). Subsequent covariate‐adjusted depression likelihood by class was estimated using the Bolck et al. (2004) to account for uncertainty in class membership. The three‐step BCH approach explicitly incorporates the classification error probabilities when estimating the relationship between latent class membership and the distal outcome (Asparouhov and Muthén 2021). By doing so, the BCH method provides odds ratios for depression that are adjusted for the classification uncertainty inherent in the LCA. The final step of the three‐step approach estimated included class x covariate interactions to estimate covariate effects on the likelihood of depression within each class.

Missing data were handled with multiple imputation by chained equations with predictive mean matching (MICE). This technique treats missing values as dependent variables in regression models with all other variables in the dataset serving as independent variables, thus maximizing available information to generate unbiased estimates for missing values (Azur et al. 2011). MICE is uniquely capable of handling data collected from complex survey designs as well as large amounts of missing data (Lee and Huber 2011; Morris et al. 2014). Data management, multiple imputation, and descriptive analyses were conducted in R Version 4.4.0; latent class analysis was conducted in Mplus Version 8.10.

## 7 Results

### 7.1 Results of Latent Class Analysis

The four‐class solution emerged as the best option (Table 2). Although BIC was superior in the two‐ and three‐class solutions, AIC and entropy favored four classes. Although entropy was superior with five classes, this solution included small (*n* < 50) classes and convergence issues. Based on the combination of statistical factors as well as considerations of interpretability, the four‐class solution was selected.

**Table 2:** Comparison of the 1‐through 5‐class solutions.

Classes	AIC	BIC	Entropy
1	9,736.816	9,790.443	—
2	9,226.711	9,339.327	0.685
3^a^	9,191.580	9,363.19	0.709
**4**	**9,173.219**	**9,403.812**	**0.740**
5^a^,^b^	9,175.528	9,465.111	0.774

*Note:* Bold indicates the solution selected for the present study.

a Solution includes small class size (*n* < 50).

^b^ Model does not converge.

**Figure 1:**
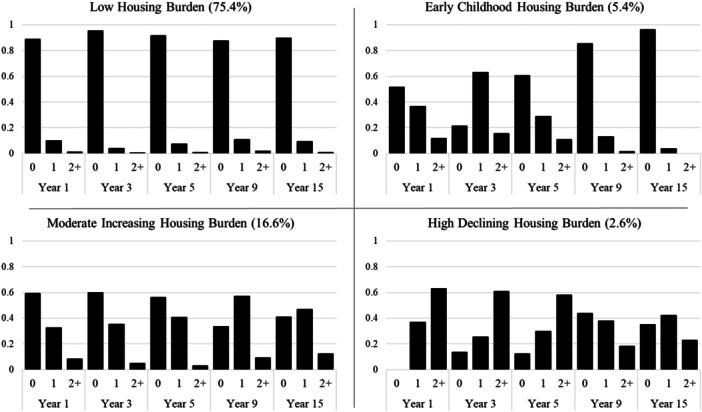
Item response probabilities by latent class membership. *Note*: Bars represent the probability of experiencing 0, 1, or 2+ housing hardships at each wave from Year 1 through Year 15.

**Figure 2:**
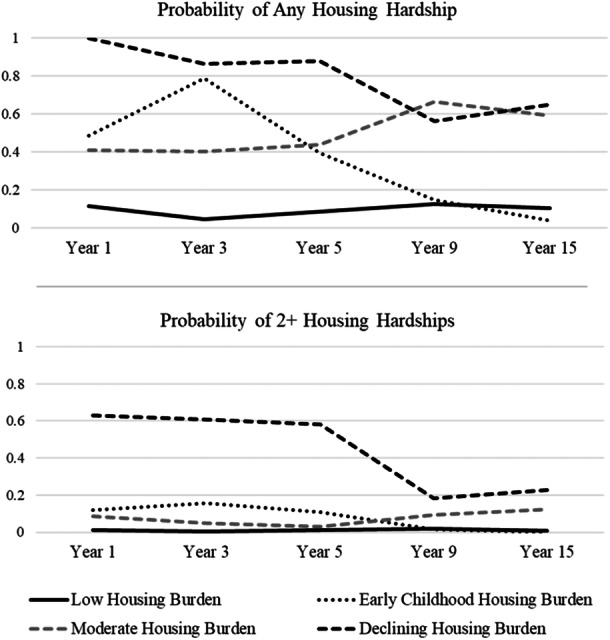
Probabilities of incidence of housing insecurity by year across four latent classes. *Note:* Figure indicates the probabilities of youth having experienced any incidence (top panel) and two or more (bottom panel) incidences of housing insecurity at each wave by latent class membership.

The four classes differed on childhood trajectories of housing insecurity (Figure 1). The first and largest class, “Low Housing Insecurity,” included youth who experienced little to no incidences of housing hardship during childhood (*n* = 1,688; 75.4%). Individuals in this class had a greater than 85% probability of experiencing no housing insecurity and a less than 2% probability of experiencing two or more incidences of housing hardship at any single wave in childhood. Youth in the second class (*n* = 121; 5.4%), “Early Childhood Housing Insecurity,” displayed comparatively elevated risk for at least one housing hardship in Years 1–5, but less than 15% probability of any housing hardship in Year 9 and a 0% probability of any housing hardship in Year 15. The third class, “Moderate Increasing Housing Insecurity” (*n* = 372; 16.6%), was characterized by consistent moderate risk for housing insecurity with a slight uptick in later waves. Children in this class faced a roughly 40% probability of any housing insecurity in Years 1–3, which increased to 67% and 60% in Years 9 and 15; furthermore, the probability of two or more hardships increased from 3% at Year 5% to 12% at Year 15. Youth in the fourth and smallest class, “High Declining Housing Insecurity” *(n* = 58; 2.6%), experienced the highest levels of housing insecurity, with a 100% probability of experiencing any housing hardship at Year 1 and a greater than 50% probability of experiencing at least one housing hardship at every other wave [Figure 2]; nonetheless, the likelihood of experiencing two or more housing hardships at a single wave declined from over 60% at Year 1% to 23% at Year 15.

### 7.2 Childhood Covariates of Latent Class Membership

Multivariate logistic regression investigated the association between covariates and likelihood class membership, with the largest class “Low Housing Insecurity” serving as the reference group (Table 3). Experiencing poverty in childhood was associated with greater likelihood of belonging to the “Early Childhood Housing Insecurity” by more than double (OR = 2.71, 95% CI = 1.22–6.023; *p* < 0.05), the “Moderate Increasing Housing Insecurity” class by more than triple (OR = 3.38, 95% CI = 1.95–5.88 *p* < 0.001), and the “High Declining Housing Insecurity” class by nearly tenfold (OR = 9.38, 95% CI = 1.97–44.60; *p* < 0.01) relative to the “Low Housing Insecurity” class. Mother's age at the study focal child's birth was associated with a slight reduction in likelihood of belonging to the “Moderate Increasing” class; each 1 year older that a mother was when giving birth was associated with 6.4% reduced odds of “Moderate Increasing Housing Insecurity” versus “Low Housing Insecurity” (OR = 0.94, 95% CI = 0.90–0.97; *p* < 0.001). Having a Black mother was associated with lower odds of belonging to the “High Declining Housing Insecurity” class relative to the “Low Housing Insecurity” class (OR = 0.242, 95% CI = 0.08–0.76; *p* < 0.01), whereas having a father who was Black or Other race was associated with substantially higher odds (OR = 12.9, 95% CI = 1.67–100.09; *p* < 0.01 and OR = 17.53, 95% CI = 1.94–158.91; *p* < 0.05 respectively), although small cell sizes for father's race in this class contributed to very large confidence intervals.

**Table 3:** Covariates of latent class membership.

	Early childhood insecurity versus low housing insecurity	Moderate increasing housing insecurity versus low housing insecurity	High declining housing insecurity versus low housing insecurity
Mother's race			
Black	0.387 [0.107, 1.394]	0.869 [0.371, 2.039]	0.242* [0.077, 0.762]
Hispanic	0.476 [0.155, 1.462]	0.671 [0.277, 1.624]	0.339 [0.094, 1.221]
Other	5.846* [1.372, 24.918]	0.000 [0.000, 0.000]	0.140 [0.013, 1.461]
Father's race			
Black	1.838 [0.450, 7.512]	2.019 [0.747, 5.462]	12.913* [1.666, 100.092]
Hispanic	1.808 [0.533, 6.130]	1.333 [0.487, 3.651]	4.632 [0.536, 40.064]
Other	0.000 [0.000, 0.000]	3.006 [0.906, 9.974]	17.534* [1.935, 158.912]
Mother's age^a^	0.978 [0.928, 1.030]	0.936*** [0.902, 0.971]	0.951 [0.892, 1.014]
Parents married^a^	0.346 [0.111, 1.084]	0.836 [0.465, 1.505]	0.954 [0.351, 2.591]
Childhood poverty	2.714* [1.223, 6.023]	3.384*** [1.946, 5.884]	9.367** [1.968, 44.595]

^a^ At child's birth.

* *p* < 0.05

** *p* < 0.01

*** *p* < 0.001

### 7.3 Emerging Adulthood Depression by Latent Class Membership

Probability of experiencing depression at the Year 22 interview different significantly across latent classes (Table 4). Youth in the “Low Housing Insecurity,” “Early Childhood Housing Insecurity,” and “Moderate Housing Insecurity” classes had similar probabilities of depression (38%, 39%, and 36% respectively) whereas those in the “High but Declining Housing Insecurity” differed significantly from the “Low” and “Moderate Increasing” classes with a 68% probability of depression at Year 22. There was no significant difference in Year 22 depression between youth in the “Early Childhood” and “High Declining” classes.

**Table 4:** Probability of emerging adulthood depression by latent class membership.

		Low housing insecurity	Early childhood housing insecurity	Moderate increasing housing insecurity
	Probability	χ^2^	χ^2^	χ^2^
Low housing insecurity	0.383			
Early childhood housing insecurity	0.391	0.012		
Moderate increasing housing insecurity	0.360	0.279	0.111	
High declining housing insecurity	0.573	4.607*	2.284	4.707*

*Note:* χ^2^ values indicate differences in probability of emerging adult depression in the comparison class (rows) relative to the reference class (columns); Table presents unadjusted probabilities by class membership.

* *p* < 0.05.

### 7.4 Covariates of Emerging Adulthood Depression

Analyses controlled for additional characteristics potentially related to emerging adulthood depression (Table 5). Female gender was associated with significantly higher levels of depression for all youth except those in the “Early Childhood Housing Insecurity” class, including a fourfold increase in risk for those in “High but Declining Housing Insecurity” (OR = 4.35, 95% CI = 1.00–18.95). Living with a parent at age 22 was protective for youth in the “High but Declining” class (OR = 0.13, 95% CI = 0.03–0.60). Working at age 22 was associated with a *higher* risk for depression among youth in the “Low Housing Insecurity” class (OR = 1.33, 95% CI = 1.07–1.66) but a substantially *lower* risk among youth in the “High but Declining Housing Insecurity” class (OR = 0.05, 95% CI = 0.01–0.27). Childhood anxiety and depression problems had likewise conflicting associations, with a slightly elevated risk for emerging adulthood depression among youth in the “Low” class (OR = 1.02, 95% CI = 1.00–1.04) but a slightly lower risk for emerging adulthood depression among youth in the “Moderate Increasing” class (OR = 0.96, 95% CI = 0.93–0.99). A history of caregiver depression and current housing insecurity—housing hardship experienced by emerging adults at the Year 22 interview—were associated with depression among those in the “Low Housing Insecurity” class only (OR = 1.50, 95% CI = 1.20–1.86 and OR = 1.67, 95% CI = 1.25–2.22, respectively).

**Table 5:** Covariates of emerging adulthood depression.

	Low housing insecurity	Early childhood housing insecurity	Moderate increasing housing insecurity	High declining housing insecurity
	OR [95% CI]	OR [95% CI]	OR [95% CI]	OR [95% CI]
Female gender	1.942*** [1.574, 2.395]	1.941 [0.976, 3.859]	2.942*** [1.849, 4.681]	4.354* [1.001, 18.948]
Lives with parent	0.973 [0.786, 1.204]	0.935 [0.455, 1.920]	1.115 [0.703, 1.769]	0.132** [0.029, 0.601]
Currently working	1.329* [1.065, 1.658]	1.481 [0.747, 2.934]	0.691 [0.429, 1.113]	0.050*** [0.010, 0.265]
Currently in school	1.462** [1.166, 1.833]	1.792 [0.784, 4.094]	1.764* [1.040, 2.993]	1.720 [0.258, 11.484]
Current housing insecurity	1.836*** [1.464, 2.302]	1.076 [0.542, 2.137]	1.194 [0.869, 1.640]	1.937 [0.910, 4.124]
Caregiver depression	1.495** [1.203, 1.858]	1.797 [0.518, 6.232]	1.142 [0.781, 2.661]	3.831 [0.002, 7,565]
Childhood depression	1.020* [1.003, 1.037]	1.015 [0.966, 1.066]	0.962* [0.932, 0.994]	0.988 [0.911, 1.072]

* *p* < 0.05

** *p* < 0.01

*** *p* < 0.001

### 7.5 Alternative Analytic Approaches

Additional RMLCA models were estimated with both a dichotomous (any housing insecurity) and continuous (numeric count of housing insecurity experiences) measure. Ultimately, poor model fit and convergence issues prevented pursuit of these efforts.

## 8 Discussion

Depression among emerging adults is a growing problem with important implications for long‐term well‐being. Childhood housing insecurity is a known risk factor for subsequent depression, but little research has examined divergent trajectories of childhood housing insecurity and their associations with emerging adulthood depression. The present study examined childhood trajectories of housing insecurity and implications for subsequent emerging adulthood depression in a large sample of families across the U.S. Findings suggest elevated depression risk associated with early, severe, and persistent housing insecurity.

Repeated measures latent class analysis identified four distinct trajectories of housing insecurity experienced by a sample of children born in 20 large American cities from age 1 to age 15; the majority, 75%, experienced consistently low levels of housing insecurity across childhood, whereas three smaller classes experienced housing insecurity at varying frequencies and developmental timings. Youth in the “Early Childhood Housing Insecurity” class experienced housing insecurity in early childhood but stabilized after age 5. The “Moderate Increasing Housing Insecurity” class experienced moderate levels of housing insecurity that increased slightly in later waves. Finally, a small class, “High but Declining Housing Insecurity,” experienced high levels of housing insecurity in early childhood that improved over time, but nonetheless remained higher than other classes on average through adolescence. Childhood poverty was associated with increased likelihood of belonging to one of the three housing insecure classes relative to the “Low Housing Insecurity” class, and having a nonwhite father was associated with increased likelihood of belonging to the “High but Declining” class.

Analyses further investigated whether risk for depression in emerging adulthood differed by childhood housing insecurity trajectories. Youth in the “High but Declining Housing Insecurity” class, who had the highest levels of housing insecurity across childhood despite improvement over time, displayed significantly increased risk for emerging adulthood depression relative to the “Low” and “Moderate Increasing” classes, which did not differ from each other in depression risk. The “High but Declining” class was the only group to ever experience a greater than 50% probability of two or more housing hardships in a single wave, which occurred in Years 1–5; this frequency of housing insecurity in early childhood may be particularly harmful to long‐term mental health, regardless of relative improvement over time. Youth in the “Low Housing Insecurity” class were not any lower risk for emerging adulthood depression than youth in the “Early Childhood Housing Insecurity” and “Moderate Housing Insecurity” classes, suggesting that only very severe housing hardship in early childhood that does not fully resolve through adolescence elevates risk for long‐term mental disorder.

Youth in the “Early Childhood Housing Insecurity” and “High but Declining Housing Insecurity” classes displayed similar general trends of elevated risk for housing insecurity in Years 1–5 that were eventually reduced in middle childhood and adolescence. Although youth in the “High but Declining” class continued to experience elevated levels of housing insecurity that were more than 50% greater than those experienced by the “Early Childhood” class at ages 9 and 15, the two groups did not differ significantly on risk for depression at age 22. Findings converge with earlier research pointing to early childhood as a uniquely vulnerable period during which socioeconomic hardship is particularly damaging long‐term (Hatem et al. 2020; Rumbold et al. 2012); the current study suggests that early childhood housing insecurity, even if resolved, may be consequential for long‐term mental health.

In addition to variation by housing trajectories, emerging adulthood depression was further associated with youth characteristics. Female gender was associated with increased emerging adult depression in three of the four housing insecurity classes. Women in general report depression symptoms at higher rates than men (Greenberg et al. 2021)—a finding that has been replicated in samples of emerging adults (Galambos et al. 2006) and held true in the present study across differing levels of childhood housing insecurity. Physiological, cultural, and socioeconomic explanations have been proposed for this phenomenon (Albert 2015), although emerging adult women may face unique vulnerabilities. Emerging adulthood is a high risk period for IPV victimization (Gracia et al. 2023; Sanz‐Barbero et al. 2018), which most commonly affects women and is a strong risk factor for depression (Breiding 2015; Matud et al. 2023; Shen and Kusunoki 2019). Nearly half of female IPV survivors (45.2% or 27.5 million) are first victimized in emerging adulthood (Leemis et al. 2022). Women are also more likely than men to experience poverty across adulthood, with one of the biggest gender disparities occurring between men and women age 18 to 34 (Bleiweis et al. 2020). Therefore, emerging adult women may face particularly elevated risk for depression compared to men and other age groups.

Working, attending school, and experiencing current housing insecurity were associated with higher risk for depression only among youth who had experienced little to no housing insecurity in childhood; these youth, who were more socioeconomically privileged than those in other classes, may have been more likely to be both working and attending college at age 22, thus experiencing greater stress that led to depression. Youth in this class were also the only ones to experiencing elevated risk for depression associated with caregiver depression or childhood anxiety or depression. It is possible that genetic predisposition for depression emerged as the most salient threat to mental health in the absence of significant environmental stressors for this class. Analyses did not distinguish between which primary caregiver experienced depression, although most were mothers. It is possible that a genetic link between maternal depression and female emerging adult depression further explains gender disparities in findings. For youth with little to no prior exposure to housing insecurity, it is possible that the new onset of housing problems in the transition to adulthood triggered extreme psychological distress relative to other youth. Future research should examine these questions, as well as the impacts of specific combinations of work and schooling during this period.

Findings have important developmental and policy implications from infancy through emerging adulthood. First, the diversity of housing trajectories that emerged among children points to a need for early and ongoing assessment of housing status for families, as well as improved widespread access to preventative services. Early identification of housing warning signs such as cost burden or missed rent payments by pediatricians or daycares may help families access light‐touch interventions that prevent short‐term hardship from becoming major housing crises. Most eviction judgments are for roughly 1 months' rent (Urban et al. 2019), e.g., so small amounts of financial assistance for families faced with eviction filings have the potential to prevent displacement and keep children housed. Extension of COVID‐era housing protections such as eviction moratoria and emergency rental assistance funds hold promise for reducing housing insecurity and homelessness long‐term (Ali and Wehby 2022; Marçal et al. 2022; McCarty et al. 2021). Likewise, preventative mental health supports for insecurely housed children embedded in schools or state early intervention programs may buffer effects of inadequate housing (Guralnick 2011; Haskett et al. 2016). Emphasizing supports for the highest risk families with very young children under age 5 may offer the potential to prevent substantial burden of mental disorder in the transition to adulthood.

### 8.1 Limitations

Findings must be understood in the context of study limitations. First, FFCW sampled an exclusively urban sample of families (Reichman et al. 2001); therefore, results cannot be generalized to children born in suburban or rural areas, who may experience different trajectories of housing insecurity. Second, the long‐running FFCW study included some attrition; approximately one in three children originally sampled did not participate in the Year 22 follow‐up survey, although the baseline and Year 22 samples were similar on key demographics (Princeton University 2024). The analytic sample was further limited to those children who remained in at least the partial custody of a biological parent across childhood in order to account for children's exposures to housing hardships across all waves. Although this included the majority of children in the sample, it risked excluding some of the most vulnerable who may have experienced changes in primary caregivers, foster care placements, and other informal caregiving arrangements. Next, due to data limitations and temporal ordering concerns, caregiver depression was not assessed as a covariate of housing trajectory class membership; prior research suggests that maternal depression increases risk for housing insecurity, and thus may have emerged as a significant predictor of class membership in the present study. Finally, the measure for housing insecurity may not have captured the full range of potential housing hardships and did not distinguish between types of housing hardships. Future research should examine unique effects of childhood eviction and homelessness trajectories on long‐term mental health and other outcomes.

## 9 Conclusions

Repeated exposure to housing insecurity in childhood poses a long‐term threat to mental health. Emerging adults with a history of early and ongoing housing insecurity in childhood face elevated risk for depression, particularly if they are women facing current housing insecurity as well. Given the importance of emerging adulthood for ongoing adult mental health, findings emphasize the long‐term developmental significance of early adversity and the potential cascade of developmental implications. At the same time, the delayed nature of the association between housing insecurity and depression highlight opportunity to promote a positive adult mental health trajectory. Early screening and supports for families with infants and young children offer promise to reduce the burden of mental disorder in the transition to adulthood.

## Conflicts of Interest

The author declares no conflicts of interest.

## Ethics Statement

The Future of Families and Child Wellbeing Study was approved by the Princeton University and Columbia University Institutional Review Boards and conforms to the Declaration of Helsinki and US Federal Policy for the Protection of Human Subjects.

## Data Availability

The data that support the findings of this study are openly available in Inter‐university Consortium for Political & Social Research at https://www.icpsr.umich.edu/web/DSDR/studies/31622/versions/V3.
